# Polyurethane-Based Gel Electrolyte for Application in Flexible Electrochromic Devices

**DOI:** 10.3390/polym14132636

**Published:** 2022-06-28

**Authors:** Christopher Johannes, Michael Hartung, Hans-Peter Heim

**Affiliations:** Polymer Engineering, Institute of Material Engineering, University of Kassel, 34125 Kassel, Germany; hartung@uni-kassel.de (M.H.); heim@uni-kassel.de (H.-P.H.)

**Keywords:** electrolyte, electrochromic device, flexible

## Abstract

For the application in flexible electrochromic devices (ECDs) on plastic substrates, a new polyurethane-based gel electrolyte was manufactured. In this context, the curing behavior and the influence of the proportion of solvent and salt on the ion conductivity as well as the optical and mechanical properties were investigated. Furthermore, the stoichiometric ratio of the polyurethane matrix was varied to influence the ion conductivity. As an isocyanate component, the aliphatic difunctional polyisocyanate prepolymer, synthesized by Hexamethylen-1,6-diisocyanat (HDI), was chosen since the resulting polyurethane is considered to be particularly lightfast, color-stable and temperature-resistant and therefore frequently used for paints and coatings. As polyol a trifunctional polyetherpolyol was selected to form a wide-meshed crosslinked matrix to achieve a mechanically stable but flexible electrolyte, that enables the processing and bending of film-based ECDs. The additives amount and the matrix stoichiometric ratio affected the curing behavior and curability. The salt content had almost no influence on the measured properties in the chosen experimental space. Solvent content had a great influence on ion conductivity and mechanical properties. An understoichiometric ratio of the polyurethane matrix (0.85) increases the ion conductivity and the mechanical flexibility, but also the optical properties in a negative manner. The best specific ion conductivity with 10^−5^ S/cm was reached with an understoichiometric ratio of 0.85 and a high solvent content (30 wt%). Concluding, due to its high flexibility and transmittance, color neutrality and sufficient ion conductivity, the application of the researched electroyte in ECDs might be suitable. A demonstrator ECD was successfully manufactured and conducted.

## 1. Introduction

### 1.1. Motivation

An electrochemical cell consists of electrodes connected by or separated by the electrolyte. If chemical reactions take place voluntarily in the electrode space between the electrodes and these generate current, the cell is a galvanic cell. If chemical reactions are forced by an external current source, it is an electrolytic cell [1].

Electrolytes play a crucial role in the properties of the systems and are used in all those applications that involve an electrochemical cell. Some important ones are accumulators, capacitors, fuel cells, sensors and actuators, solar cells and electrochromic systems [2]. Systems with electrochromic function are characterized by the fact that they reversibly change their transmission properties in the visible spectral range when DC electrical voltages are applied. These are electrochemical cells within which a redox reaction takes place, changing the color of the electrochromic material. This makes the integration of this function particularly interesting in the window glazing of buildings or vehicles for light and heat regulation. In addition to applications in architectural glazing, products have also been known for some time in other areas, e.g., as rear-view mirrors in automobiles (glare protection), as simple segment displays for price marking (display) or as eye protection (ophthalmic). What these systems have in common is that they are largely rigid. However, there is now a great demand for flexible systems, e.g., films with electrochromic properties that can be subsequently applied to glazing, or in very innovative areas of technology such as “wearable” displays or sensors that require high reversible stretchability because, among other things, they need to be foldable [3]. The further processing of film-based flexible systems in plastics engineering processes such as thermoforming or injection molding for the production of flat, three-dimensional components with electrochromic function could also be of interest, for example in automobiles. Essential requirements for the electrolyte were derived from these uses. It must be mechanically stable, but flexible and ductile. Due to the optical application, a high transmittance and colorlessness are important. A good ionic conductivity of 10^−7^ to 10^−3^ S/cm at room temperature is crucial to minimize the voltage drop across the device and to realize acceptable switching times [4]. Polymer electrolytes are particularly suitable for these requirements because they are dimensionally stable but generally flexible in contrast to non-polymeric inorganic electrolytes, do not tend to leak like liquid systems and can absorb or transmit forces due to their strength [5,6,7,8,9,10,11,12]. Their chemical resistance is also higher and so-called dendrite growth [13] at the electrodes (in accumulators) is suppressed [14,15]. In addition, they are easier and less expensive to manufacture and process, and bonding to the electrodes is generally better due to deformation and adhesion properties. Solid polymer electrolytes are furthermore being intensively researched because they are, in contrast to the mostly liquid electrolytes that have been used up to now, non-flammable, do not build up hydrostatic pressure and thus increase the safety of applications, especially those that require a large quantity of electrolyte, such as electric vehicles with their large accumulators that are often distributed throughout the vehicle. The main weakness is the lower ionic conductivity.

### 1.2. Classification of Polymer Electrolytes

Several proposals are made in the literature for the classification of electrolytes. Frequently, a distinction is made with regard to the aggregate state (solid or liquid) and the substance (organic or inorganic). Polymer electrolytes can be liquid, solid or—as an intermediate form—gel-like, as well as organic (i.e., with carbon as an essential main chain component, e.g., polyethylene oxide) or inorganic (i.e., without carbon as an essential main chain component, e.g., polysiloxane). Generally, polymer electrolytes are understood as those electrolytes consisting of a polymer matrix with salt mixed in and characterized by significant ionic conductivity [16]. The mixed-in salt is solvated and is then present in ionized form as a cation and an anion. In principle, these can then act as mobile charge carriers for the desired ion transport. Following [2,4], a distinction is usually made between “solid polymer electrolytes” (SPEs), “gel polymer electrolytes” (GPEs), “polyelectrolytes” and “composite polymer electrolytes” (CPE), although the definition or delimitation is not always uniform. For example, in [4] the resulting electrolyte is referred to as GPE when a liquid plasticizer or solvent is incorporated into a polymer matrix and a stable gel is formed. In [2], on the other hand, an electrolyte is referred to as a “plasticized polymer electrolyte” (PPE) after adding small amounts of solvent or ionic liquids to other polymer electrolyte classes. SPEs are defined as solvent-free systems consisting of a polar polymer matrix with salt dissolved in it. In addition to electrically neutral repeating units, polyelectrolytes also consist of ionized units, i.e., covalently bonded charged groups. If inorganic fillers or ionic liquids are additionally added to the GPEs, they are called CPEs [4].

### 1.3. Transport Mechanism in Polymer Electrolytes and Influencing Factors

This essentially results from the transport mechanism of ions in polymers. The accepted model assumes moving polymer chains or segments carrying regular polar or ionic groups. These groups interact with the ion, which is usually positively charged and metallic. This interaction, the externally imposed electric field and the polymer chain movements can result in transport of the ion through the electrolyte. In this case, the transport is described as discontinuous, as jumping (“hopping”) from one polar or ionic group to another within a polymer chain or between polymer chains. Ion clusters consisting of the dissolved cations and anions (unless they are polyelectrolytes with anions covalently bound in the polymer chain and only freely mobile cations) can also contribute to transport in this model [11,17,18].

Against the background of the described transport mechanism of ions in polymers, it can be deduced that influencing variables that lead to more and less strongly bound mobile charge carriers and to better mobility of the polymer chains have a positive influence on conductivity. Temperature generally has a strong positive influence on conductivity. At higher temperatures, the polymer chains are more mobile, and the molecular distances are larger or secondary valence forces are smaller. The resistance for ionic conduction decreases. The relationship can often be described by the Arrhenius Equation (1) [6,7,19]:

(1) $$\sigma =\sigma_{0}^{\left(\frac{-e_{a}}{k\times T}\right)}$$

with the temperature *T*, the conductivity at 0 °C *σ*_0_, the Boltzmann constant *k* and the activation energy *e_a_*.

Furthermore, the so-called Vogel–Fulcher–Tammann equation (VFT) (2) describes in good approximation the temperature dependence of the conductivity of SPEs and was developed for pure polymer systems:

(2) $$\sigma =\sigma_{0}^{\left(\frac{-e_{a}}{T-T_{0}}\right)}$$

Here, *T*_0_ is a reference to the glass transition temperature (*T_g_*) of the polymer material, often about 50 K below *T_g_* [20]. From this context, it can be concluded that it is advantageous for conductivity if the polymer has a low *T_g_* or if the application temperature is significantly above it. This is because at or below *T_g_*, SPEs exhibit almost no ionic conductivity, which is plausible in light of the transport model described. At the glass transition, the polymer chains “freeze,” i.e., the mobility of the chains decreases abruptly and the ions are “held” between the chains. A similar phenomenon and negative influence is present in crystallized material. In crystalline structures, the polymer chains have a high degree of order, i.e., they are oriented in the same way and are not entangled as in the amorphous state. At the same time, the secondary valence forces are high, the free volume is low and the mobility of the chains is very limited. For example, polyethylene oxide, as one of the intensively researched materials for SPEs, shows a higher ionic conductivity with increasing proportion of amorphousness, respectively decreasing proportion of crystalline regions with increasing temperature. Only at above 65 °C does the amorphous phase dominate and a conductivity of ≥10^−4^ S/cm can be achieved [9]. Adding plasticizers or solvents has a positive effect, because it leads to a reduction of secondary valence forces, larger free volume, better chain mobility and thus better ionic conductivity. A higher salt concentration leads to a higher number of charge carriers and thus tends to increase the conductivity (e.g., [7]). However, above a certain concentration, the conductivity decreases again because the ions lead to a physical crosslinking of the polymer chains and thus to a reduction in mobility and an increase in *T_g_*. Accordingly, there is often an optimal salt concentration for the ionic conductivity [5,19,21]. The salt used, or the cation and anion bound in it, also have a major influence on conductivity. For example, such electrolytes with lithium cations show significantly higher ionic conductivity compared to other metal cations such as sodium or aluminum, whereas smaller ions (i.e., measured by their nucleon number) tend to lead to better conductivity [22]. Furthermore, there is an influence by the type of anion: Anions with delocalized electron densities thus enable high ionic conductivities, because the delocalized charge promotes the dissolution of the ion pair and leads to a higher concentration of free cations (e.g., (4-styrenesulfonyl)(trifluoromethanesulfonyl) imide (STFSI)) [22]. In addition, it was observed that larger anions have a “plasticizing” effect, i.e., they reduce the secondary valence forces of the polymer chains and increase the mobility and thus the conductivity [2].

### 1.4. Framework of Present Study

Polyethylene oxide (PEO) is a commonly used, early and intensively researched matrix material in polymer electrolytes due to its highly polar ether bonding to coordinate the cation (usually lithium cation) [2,11,16], whereby the properties are strongly dependent on the molecular mass, i.e., the polymer chain length [23]. The mechanical strength tends to improve with increasing chain length due to the increase in the degree of crystallization, with the ionic conductivity tending to decrease. Conversely, ionic conductivity is greater with higher amorphous content due to shorter chain lengths, with mechanical strength decreasing, e.g., in [5,9] it is described that pure PEO has a good ionic conductivity of <10^−4^ S/cm only above 60 °C, with the shear modulus decreasing significantly in this range. At room temperature, the ionic conductivity is lower by up to three orders of magnitude.

Different approaches have been taken to improve the properties (see reviews [2,12,17,21,24]). Comparatively simple measures include the addition of plasticizers and solvents, the use of branched or crosslinked molecules to avoid crystalline structures [25,26] as well as the blending with other polymers [6,27,28,29] or the introduction of fillers [30,31,32,33]. Other amorphous or weakly semi-crystalline polymers are possible as matrix material as well, e.g., polymethylmethacrylat (PMMA) or polycarbonate (PC) [11,21]. Polyurethane (PU) is an interesting matrix material due to its microstructure of hard and soft segments, where the isocynate component represents the hard segment and gives mechanical strength, and the polyol component represents the soft segment that contributes flexibility. Polyetherpolyols are considered to enhance the ionic conductivity due to the cation coordinating effect of the ether bonding [6,7,19,34,35]. Bao et al. researched a solid electrolyte with PU and a weight portion of 30% LiTFSi with an ion conductivity of 3.7 × 10^−7^ S/cm at 25 °C, a tensile strength of 4.2 MPa and 987% elongation at break. Blended with PEO and the same salt content the conductivity reached 10^−6^ to 10^−5^ S/cm (1.6 MPa tensile strength, 200% elongation at break) [6]. Cong et al. studied an electrolyte with linear, non-crosslinked polyurethane molecules that formed a semi-crystalline structure, where the higher the salt content, the lower the crystallinity. With 20% LiTFSi the conductivity was 4.1 × 10^−5^ S/cm, but the tensile strength only 0.5 Mpa and the elongation at break 300% [7].

In the present work, a new polyurethane-based gel electrolyte is used and characterized, rather from a materials engineering than an electrochemical perspective. So, not only were the manufacturing process, curing behavior and optical and mechanical properties investigated, but also the ionic conductivity. The system is interesting for the following reasons, especially with regard to the above-mentioned requirements associated with ECDs. The polyurethane is a cold curing cast elastomer, which should make the preparation and application to the ECDs easy. The chosen isocynate component is an aliphatic difunctional polyisocyanate prepolymer, synthesized by HDI, since the resulting polyurethane is considered to be particularly lightfast, color-stable and temperature-resistant and therefore frequently used for paints and coatings [36]. As the polyol component, a trifunctional polyetherpolyol was chosen to form a wide-meshed crosslinked matrix to achieve a mechanically stable but flexible electrolyte, that enables the processing and bending of film-based ECDs. Beyond this, an interesting fact is that with a surplus of polyol, an increase in ionic conductivity is expected, since unbound and thus more mobile polymer chains should be present. Next to lithium salt (LiTFSi) with its small lithium cation and big anion (and their above-mentioned advantages) the regularly employed and cheap solvent propylene carbonate was used to enhance the supposed intrinsic low ionic conductivity. Because of the described influence of salt and solvent concentration in literature, the influence of the proportion of solvent and salt on the properties of this electrolyte was investigated. Furthermore, the stoichiometric ratio of the polyurethane matrix was varied to observe the influence on the ionic conductivity.

## 2. Materials and Methods

**Figure 1:**
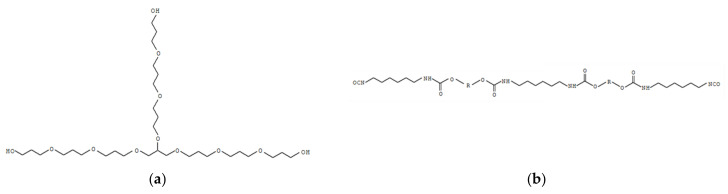
Schematic and exemplary representation of the polyurethanes basic components (**a**) polyetherpolyol and (**b**) diisocyanate, each with a possible length derived from the OH number and NCO content, respectively.

The electrolyte consists of a wide-meshed crosslinked polyurethane (PUR) matrix to which the conductive salt lithium trifluoromethanesulfonate (LiTFSi), the solvent propylene carbonate, both fromSigma-Aldrich (St. Louis, MO, USA), and a curing catalyst dibutylzinndilaurat (DBTL) from Merck KgaA (Darmstadt, Germany) were added. The polyurethane matrix was synthesized from the trifunctional polyetherpolyol Desmophen 28HS98 (OH component) and the diisocyanate Desmodur XP 2617 (NCO component) from Covestro (Leverkusen, Germany). It is classified as a cold curing polyurethane cast elastomer. The polyol with an OH number of 233 is present as a clear, colorless, viscous liquid and is reacted by propoxylation of the trivalent alcohol glycerol (Figure 1a). The isocyanate component is an aliphatic divalent polyisocyanate prepolymer with an NCO content %NCO of 12.5% synthesized from hexamethylene-1,6-diisocyanate (HDI) (Figure 1b). The HDI is not present as a monomer (<0.5 wt%), but synthesizes to an oligomeric, largely linear prepolymer and presents as a clear, colorless, viscous liquid.

The basic reaction here is the addition reaction of an isocyanate group (NCO) with a hydroxyl group (OH) to form the characteristic urethane group (Equation (3)). Since the polyol is a branched molecule with three terminal hydroxyl groups, a three-dimensionally crosslinked matrix results. Due to the length of the polyol molecules and diisocyanate prepolymer, a wide-meshed crosslinking results, that gives the material its elastomeric properties.

(3) $$R^{1}–N=C=O+HO–R^{2}\;\to \;R^{1}–NH-CO-O-R^{2}$$

The electrolyte was prepared by taking into account the stoichiometric quantitative ratio of the OH and NCO components (m_OH_/m_NCO_) for the polyurethane matrix using the formula

(4) $$m_{OH}/m_{NCO}=\left(17\times \%NCO\right)/\left(42\times 1/33\times \left(OH-\mathrm{number}\right)\right)$$

with the component’s characteristic values NCO content (%NCO) and OH number, and by adding certain parts by weight (wt%) of solvent and lithium salt. In this study, the stoichiometric quantitative ratio of the polyurethane and the parts by weight of the solvent and salt were varied (see Table 1). The solvent and lithium salt were stirred into the OH component with a magnetic stirrer while heated above 100 °C in order to dissolve the salt (dissociate into ions through solvation) and to evaporate water. After adding the NCO component and homogenizing, the mixture was evacuated in a vacuum oven (Heraeus, Germany) at 40 °C to reduce the stirred air and thus also the ambient humidity. Water can react with the NCO component to form carbon dioxide (CO_2_) and lead to undesired bubble or foam formation during the curing phase. During storage and processing it must therefore also be taken into account that the components are hydrophilic. The catalyst was stirred in the mixture for half a minute and, considering pot life, the still flowable electrolyte was processed.

**Figure 2:**
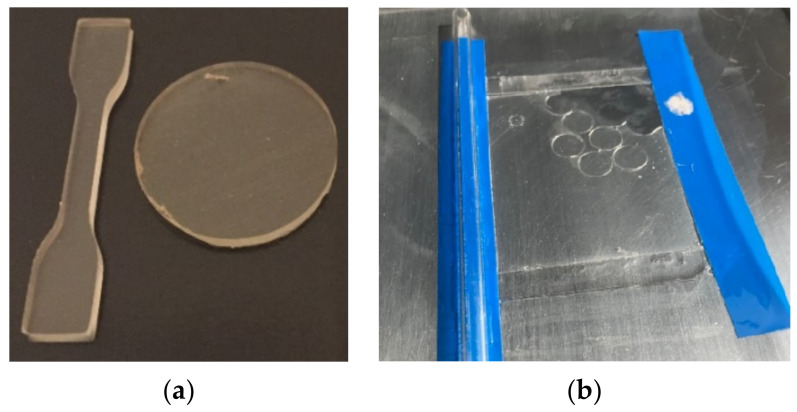
Electrolytes samples: (**a**) S3A type samples according DIN 53504 for tensile test (**left**) and Ø30 mm rounds for hardness and optical test (**right**); (**b**) Samples of around 130 µm thickness and Ø11 mm were obtained by doctor blade method for EIS.

For the mechanical and optical characterization, the electrolyte was casted in an aluminium casting mold to form a 2 mm thick plate of approximately 120 × 120 mm^2^. The mold was put in an oven at 40 °C for 22 h. After demolding of the cured, elastomeric plate, samples were punched out (Figure 2a). For electrochemical impedance spectroscopy a thin electrolyte film (~130 µm) was obtained by doctor blade method with 175 µm films and a glass rod (Figure 2b). The plate had around 100 °C for a fast curing. Samples of Ø11 mm were punched out. The usual total volume of the mixture was 65 mL to fill the casting mold and prepare the films.

Table 1 gives a summary of the electrolyte formulations used and the curability at 40 °C and 100 °C. All formulations were curable at 100 °C applying doctor blade method, but formulations h–j were not curable at 40 °C to manufacture the samples for mechanical and optical testing (see Section 3.2). The cured sample plate of mixture g with a surplus of diisocyanate showed many small bubbles all over the material, so that a tensile test was not possible. The reason for that might be the well-known polycondensation reaction of isocyanate with water forming CO_2_. This reaction is often used to foam material in polyurethane chemistry. The water could possibly come from the atmosphere during the manufacture and curing process or could be contained in the raw materials. The polyol, salt and solvent are hygroscopic. This is why all mixtures were heated up above 100 °C to evaporate the water before adding isocyanate. This could even be observed, because water condensed at the beaker around that temperature.

Consequently, an experimental design with formulations a–f and four repetitions was created to determine the properties and investigate the influence and interaction effect of salt and solvent content and the influence of a substoichiometric ratio of the polymer matrix. Due to the four repetitions, a possible batch/manufacturing influence could be examined as well. For all other formulations only the specific ionic conductivity was measured and given for reasons of comparison and interpretation.

**Figure 3:**
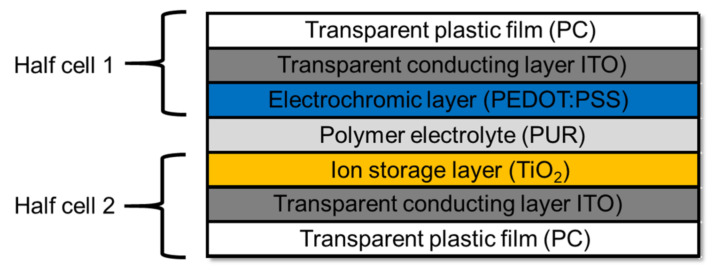
Configuration of the demonstrator ECD.

The ECD demonstrator was based on polycarbonate film (500 µm) from Covestro (Leverkusen, Germany). Indium tin oxide (ITO) was used as a transparent conducting oxide and sputtered on the substrate sheets (DIN A3) by Nanogate GfO Systems GmbH (Schwäbisch Gmünd, Germany) with a specified resistance of 25–35 Ω·sq (Elamet Trans B). As a counter electrode, titanium dioxide (TiO_2_, sheet resistance: >1 × 10^5^ Ω·sq) was coated in a sol-gel process by Fraunhofer ISC (Potsdam, Germany). The electrochromic material PEDOT:PSS (Clevios F AS) from Heraeus (Hanau, Germany) was dipcoated and applied as a working electrode. The electrolyte was dropped on the counter electrode, the working electrode was put on top. Weighted down with a weight it was put in a pressure tank at 0.4 MPa and 40 °C for 22 h in order to cure the electrolyte (Figure 3).

The Rubber Process Analyser D-RPA 3000 from MonTech Werkstoffprüfmaschinen GmbH (Buchen, Germany) was used to characterize the crosslinking behavior of the electrolytes PUR matrix. The sealed test chamber with the test material inside consists of an upper and a lower part. The lower part oscillates and measures the torque over time resulting from the shear resistance. As a parameter, the temperature was set and regulated during testing.

The spectrophotometer UltraScan Pro from Hunterlab (Reston, VA, USA) was used to characterize the transmittance and color properties of the electrolyte. It is equipped with a xenon flash lamp to generate the standard light type “D65”. The transmittance values were determined in % (0–100%) in the spectrum from 350 nm to 1050 nm according to DIN EN IS0 13468-1 and the visible light transmittance, a single calculated value to characterize the transmittance in the visible range from 380 nm to 780 nm, was determined according to DIN EN 410. The color was characterized according to DIN EN ISO 11664-4 using coordinates in the L*a*b* color space CIE 1976 color space. The value for L* describes the position on the light-dark axis, the value for a* the position on the red-green axis and the value for b* the position on the blue-yellow axis.

The electrochemical tests were conducted with a potentiostat ‘reference 600’ from Gamry Instruments (Warminster, PA, USA). In order to measure the bulk resistance and calculate the specific ionic conductivity of the electrolyte films, they underwent electrochemical impedance spectroscopy (EIS) with a two-electrode assembly from rhd instruments (Darmstadt, Germany) with stainless steel electrodes of 8 mm diameter used. A standardized initial load of calculated 4.6 N was applied by a spring in the assembly. The frequency range was from 1 MHz to 2 Hz. The resulting Bode plots were modelled with the Levenberg–Marquardt method to determine the bulk resistance R. The specific ionic conductivity σ was then calculated by

σ = h/(R × d)(5)

where h is the samples thickness and d is the electrodes area. A three-electrode setup was used for chronoamperometry and cyclic voltammetry of the demonstrator ECD.

The tensile test was conducted with a tensile testing machine from Hegewald & Peschke Meß- und Prüftechnik GmbH (Nossen, Germany) with a 500 N load cell installed according to DIN 53504 for elastomers to determine the tensile strength σ_B_ and elongation at break ε_B._ The tests were conducted with 200 mm/s under 23 °C and 50% r.h. The shore hardness (micro shore A) according to DIN ISO 7619-1 for elastomers or thermoplastic elastomers was measured with a ‘Digitest II’ from Bareiss Prüfgerätebau GmbH (Oberdischingen, Germany) with three stapled samples of 30 mm diameter each.

Water content was measured with KarlFischer titration and the thermal analysis was conducted with a DSC Q1000 and a TGA Q500 from TA Instruments (New Castle, DE, USA).

Chemical analysis was performed using a Shimadzu IRAffinity-1S Fourier transform infrared spectrometer (FTIR) (Kyoto, Japan) with an ATR unit, which measured absorption or transmission versus wave number in the range of 600 to 3800 cm^−1^.

## 3. Results and Discussion

### 3.1. Curing Atmosphere

In a preliminary test, the influence of the manufacture and curing atmosphere on the pure polyurethane properties was examined to define the standard manufacture process. The polyurethane was first mixed and cured under normal atmosphere in the laboratory, second under inert nitrogen atmosphere in a mobile glove box (N_2_, quality 5.0, O_2_-concentration <3%, measured with an oxygen meter Orbmax from Orbitalum Tools GmbH (Singen, Germany) and third mixed in the glove box but cured at 50 mbar in a vacuum oven. The material cured in vacuum was covered all over with big bubbles, and further mechanical testing was impossible, only measuring of the water content. The other two samples only contained scattered small bubbles. It is supposed that due to vacuum the small gas bubbles grew bigger and bigger as long as the material was still liquid. Apart from that, no significant differences could be observed between the different materials (Table 2). So, concluding, for all mixtures apart from formula g with a surplus of diisocyanate, the described manufacturing process under normal atmosphere works. To conduct the experiment with g and fresh raw materials under pure inert atmosphere could be interesting, although the specific ionic conductivity is not promising compared to other mixtures.

### 3.2. Curing Behavior

**Figure 4:**
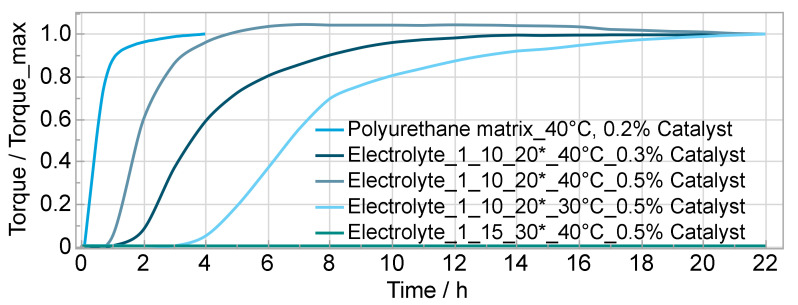
Electrolytes’ curing behavior depending on temperature, amount of catalyst and additives; * OH/NCO ratio/wt% salt/wt% solvent.

The electrolyte’s curing behavior depends on the curing temperature, the amount of additives and catalyst. Figure 4 shows the relative torque measured in the test chamber corresponding to the curing degree. The results give important information regarding the processing time of the mixture and the curing time. Adding additives or lowering the temperature prolong the curing time, adding more catalyst shortens the curing time. Obviously, at a certain weight portion of additives, the polyurethane matrix does not cure anymore at 40 °C or the polymerization is drastically slowed down, as shown in Figure 4 for the mixture j with 15 wt% salt and 30 wt% solvent. The sameg was observed for the mixtures with a surplus of polyol (stoichiometric ratio 0.85) and a weight portion of 30% resp. 35% additives (h and i). At higher temperatures it is possible to cure the material (the samples for measuring the conductivity were cured at 100 °C while being prepared with the doctor blade method). However, at 50 °C curing temperature or higher the casted electrolyte for preparing the test samples (mechanical, optical and chemical test) showed a nonuniform and uneven surface and at higher temperatures also bubbles. Because of this, and because the material is mostly cured after one day according to the results, the maximum curing temperature for all following experiments was set to 40 °C.

### 3.3. Specific Ion Conductivity

**Figure 5:**
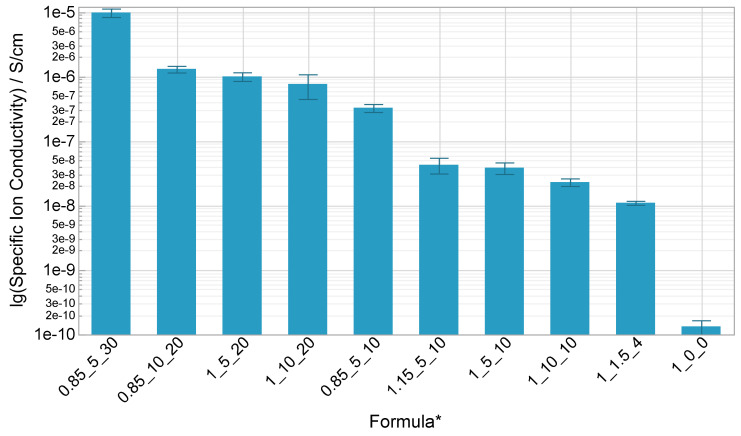
Specific ion conductivity of the electrolytes’ formulations at 25 °C on a logarithmic scale with standard error; * OH/NCO ratio/wt% salt/wt% solvent.

Figure 5 shows the mean values of the formulas’ specific ion conductivity on a logarithmic scale measured at 25 °C. The formulas 1_0_0, 1_5_10, 1_10_10, 1_5_20, 1_10_20 and 0.85_5_10 were included in the screening experiment with four repetitions and three samples each, a sample size of twelve in total. The other formula bars are each based on only three samples from the same manufacturing batch with a lower variance. The matrix itself without additives (1_0_0) has practically no conductivity. Based on the statistical analysis of the screening experiments data, the influence of the solvent content is highly relevant and significant in accordance with the literature. The mean values of the formulations with lower salt concentration (5 wt%) are higher than the mean values of the formulations with higher salt concentration (10 wt%), even if the effect is statistically not significant in the chosen experimental space. This is not inconsistent with the literature, because in several cases the conductivity decreases again with higher salt concentrations after a maximum was reached. So, an optimum salt concentration could exist between 5 and 10 wt% or below 5 wt%. An interaction effect of salt and solvent was not observed.

Using a surplus of polyol means a ratio of 0.85 compared to 1, the conductivity increases significantly by a factor of 10 (b vs. f) regarding the low factor steps (5 wt% salt and 10 wt% solvent) and by the factor of around 2 (e vs. h) regarding the high factor steps. This result seems plausible due to the better movability of the unconnected polyol molecules that foster the ion transport mechanism. A surplus of diisocynate, means a ratio of 1.15 compared to 1, has no effect on the conductivity (b vs. g). Based on the previous findings, an additional formula (i) with a surplus of polyol, low salt content and high solvent content (0.85_5_30) was tested and a specific ionic conductivity of almost 10^−5^ S/cm resulted, which is remarkably high considering the crosslinked polymer matrix and literature, even if the formula is not curable at 40 °C with 0.5 wt% catalyst.

**Figure 6:**
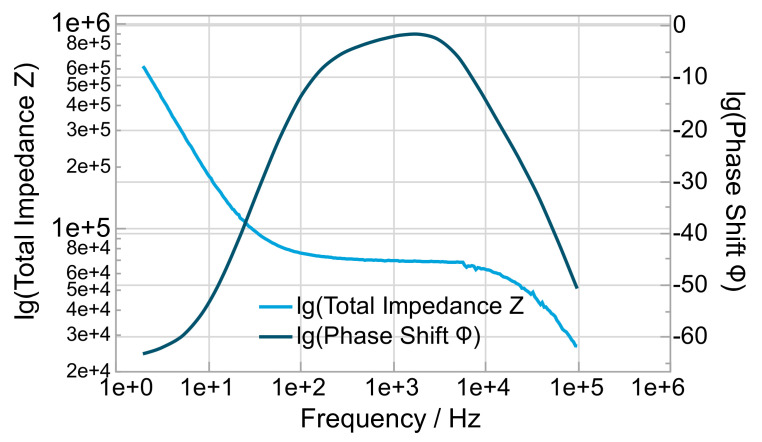
Bode plot for mixture f (0.85_5_10).

Exemplarily, Figure 6 shows the Bode plot of mixture f (0.85_5_10) with a pronounced plateau to determine the bulk resistance and calculate the specific ion conductivity by Formula (5). In the Bode plot on the primary axis, the logarithm of the total impedance lg Z, and on the secondary axis, the logarithm of the phase shift φ, are plotted versus the logarithm of the frequency lg f.

**Figure 7:**
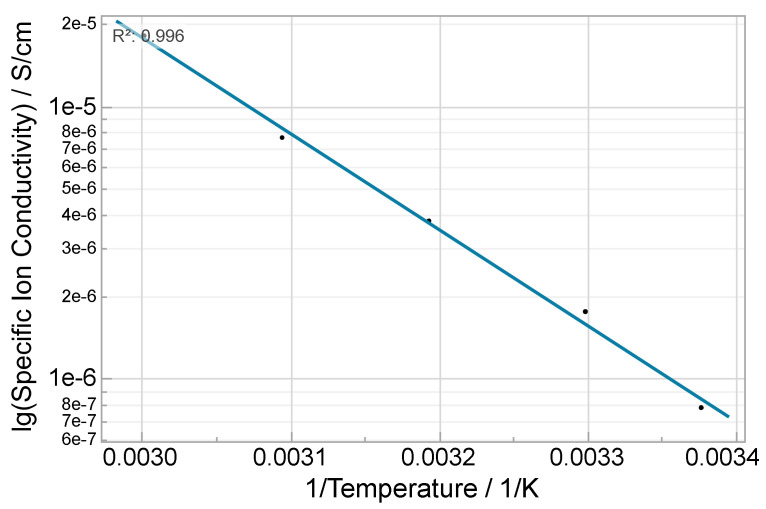
Arrhenius plot of the temperature dependent on specific ionic conductivity of an electrolyte with 15 wt% LiTFSi and 30 wt% propylene carbonate between 23 and 60 °C; adjusted R^2^ = 0.996.

The temperature dependency was researched with mixture j (1_15_30). The conductivity shows Arrhenius behavior between 23 °C and 60 °C (Figure 7), and the activation energy was calculated to 0.698 eV.

### 3.4. Mechanical Properties

**Figure 8:**
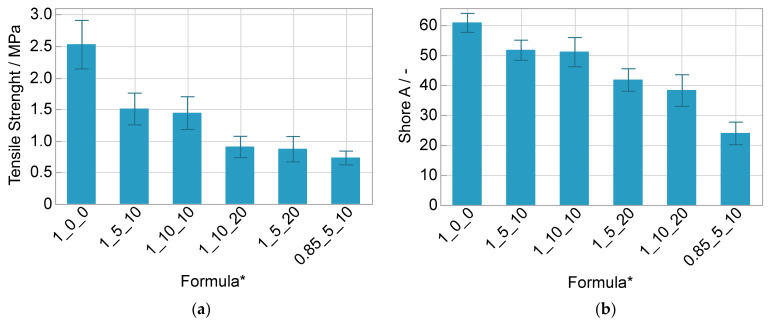
(**a**) Tensile strength (*n* = 20) and (**b**) Shore A values (*n* = 26) with standard deviation; * OH/NCO ratio/wt% salt/wt% solvent.

The solvent content has a highly significant negative effect on the tensile strength, whereas the salt content has no effect and an interaction effect could not be observed. Compared to the pure polyurethane matrix the tensile strength decreases by almost 40% at 10 wt% and by almost 65% at 20 wt% solvent content (Figure 8a). The formula with the understoichiometric ratio of the matrix (0.85_5_10) has only half of the tensile strength compared to the formula with the stoichiometric ratio (1_5_10), so it has a major significant impact. The same implications result for the hardness (Figure 8b), which is plausible because normally both properties correlate.

**Figure 9:**
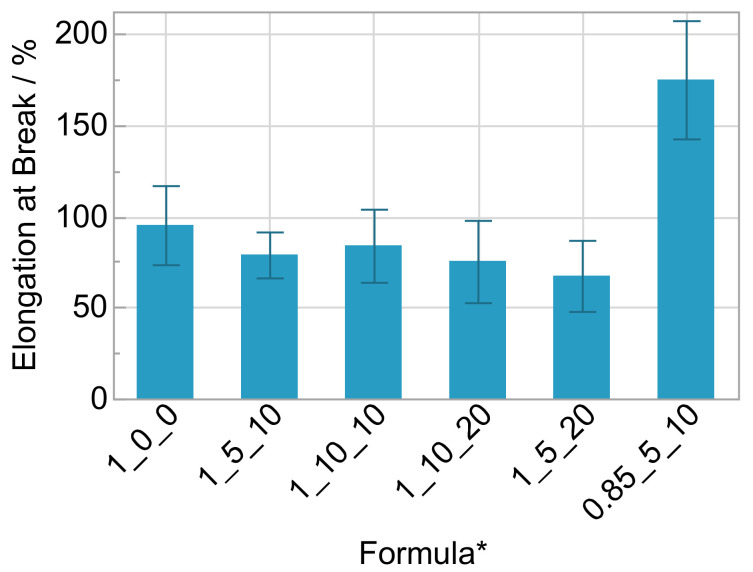
Elongation at break with standard deviation (*n* = 20); * OH/NCO ratio/wt% salt/wt% solvent.

Regarding the elongation at break, the only relevant effect is the one of the stoichiometric ratio (Figure 9). The value more than doubles with a surplus of polyol and reaches more than 170%. Decreasing tensile strength and increasing elongation at break is ascribed to the lower crosslink density of the matrix.

### 3.5. Optical Properties

**Figure 10:**
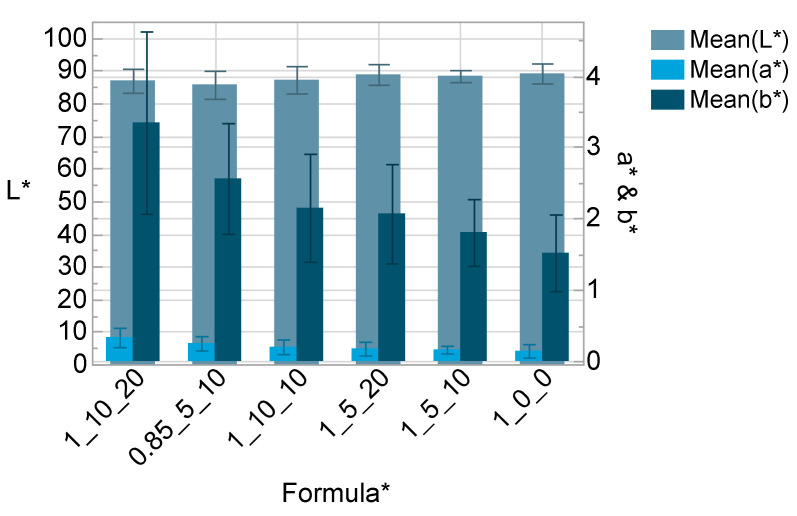
L*a*b*—Color values with standard deviation (n = 12); * OH/NCO ratio/wt% salt/wt% solvent.

Significant and relevant influence of salt and solvent content on the optical properties was only found regarding the b* color value, where the color tends to get more yellow the more additives were added, which was observable even by eye (Figure 10).

**Figure 11:**
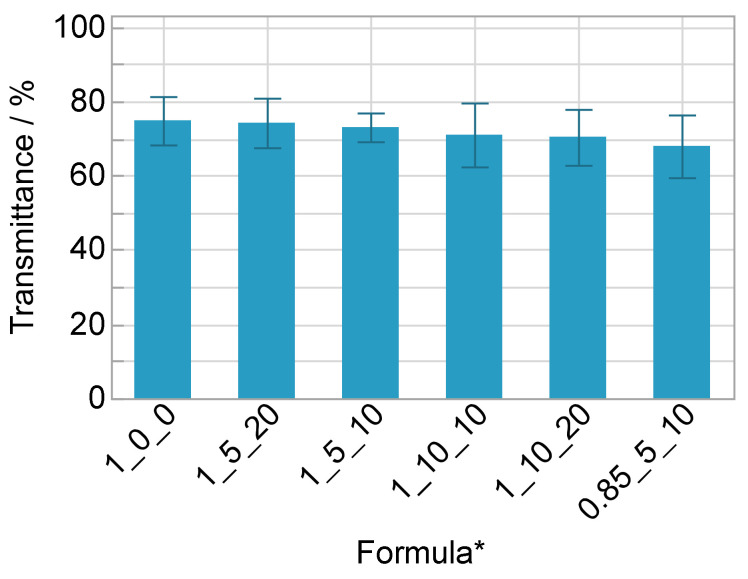
Visible light transmittance according to DIN EN 410 with standard deviation (*n* = 12); * OH/NCO ratio/wt% salt/wt% solvent.

**Figure 12:**
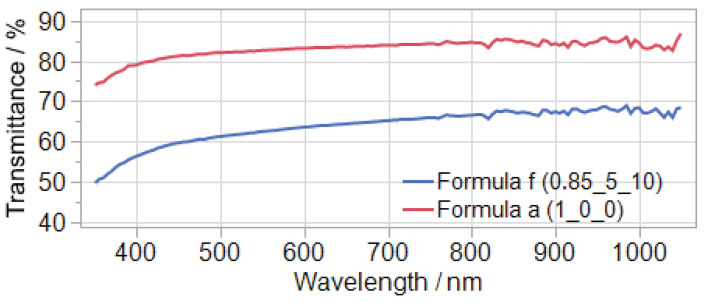
Exemplary transmittance curves of one sample of formula a and f.

The material with the substoichiometric ratio has a significant lower transmittance (Figure 11 and Figure 12) and L* value as well as a higher b* value (Figure 10) compared to the stoichiometric formula. It should be noted, that the transmittance is negatively affected by the thickness of the sample. The final electrolyte layer in the ECD is much thinner (<100 µm) than the samples measured in this study (2 mm).

### 3.6. Water Content, Thermal and Chemical Properties

**Figure 13:**
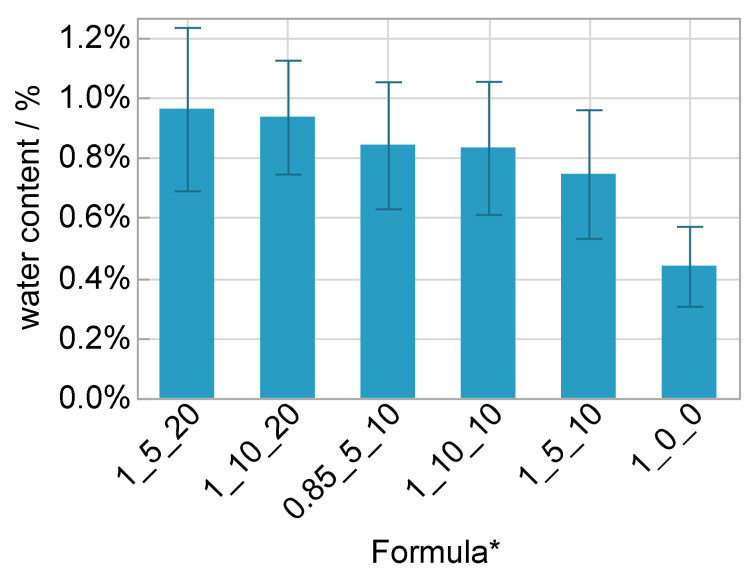
Water content with standard deviation; * OH/NCO ratio/wt% salt/wt% solvent.

The water content in all electrolyte formulations was between 0.7% and 1.0% and was higher compared to the matrix material (Figure 13). These values are significantly higher than the ones in Section 3.1. One explanation could be that fresh material was used for the atmospheric influence study. Another could be that the humidity in the laboratory at the manufacturing times differed significantly. As described above, salt, solvent and polyurethane components are hydrophilic. The more often the chemical containers were opened in normal atmosphere the higher might have been be the introduction of water. Because of this reason the mixture was heated above 100 °C and evacuated before adding the catalyst as described in Section 2. An extension of the heating and/or evacuating duration could maybe reduce the remaining water.

**Figure 14:**
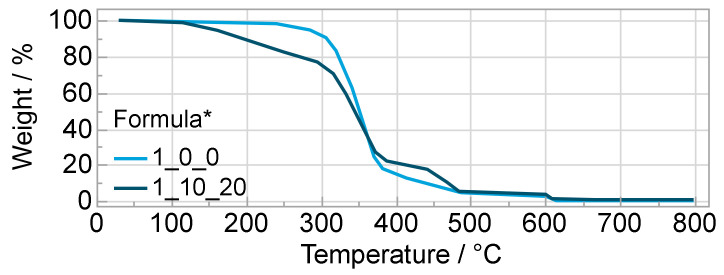
Temperature-dependent mass loss from TGA measurement of formulas* 1_0_0 and 1_10_20; * OH/NCO ratio/wt% salt/wt% solvent.

The DSC measurement showed a glass transition temperature of the polyurethane matrix at −7 to −4 °C. The TGA measurement showed a three-stage thermally induced degradation of the matrix (formula 1_0_0), where degradation processes of the first and largest stage started at about 250 °C and reached their maximum degradation rate at about 350 °C until the first stage no longer showed any significant degradation at about 400 °C with a mass loss of more than 80%. During the second step the mass reduced to 5% at 480 °C. At 600 °C the gas switched from inert gas to oxygen, which led to a quick oxidative degradation with a remaining mass of <0.2% (Figure 14).

**Figure 15:**
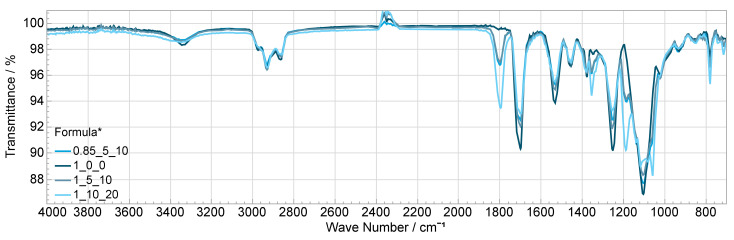
Comparison of different formulation’s FTIR curves with and without salt and solvent; * OH/NCO ratio/wt% salt/wt% solvent.

Compared with the electrolyte (Formula 1_10_20), two differences are noticeable. On the one hand, the electrolyte already loses significant mass between 100 °C and 300 °C. Since this mass loss is approximately equal to the solvent content (20%), it is assumed that the solvent volatilizes in this range (boiling point 240 °C). On the other hand, the degraded relative mass during the second stage between approximately 450 °C and 480 °C is higher in the electrolyte. Since the absolute mass of matrix and electrolyte is nearly identical from 480 °C on and a melting point of >300 °C is specified for the conductive salt, it is suggested that the salt degrades there. From Figure 15, the influences of the salt and the solvent on the FTIR spectrum can be studied. Thus, deviating at 1800 cm^−1^ and at 708 cm^−1^, one clear peak each can be attributed to the solvent propylene carbonate. The salt shows up as a band at about 1060 cm^−1^, 1130 cm^−1^, 1190 cm^−1^ and 1350 cm^−1^ [37]. The largest peak at 1100 cm^−1^ is caused by valence vibrations of the ether bonds –C–O–C– in the polyether polyol component. The peaks at 1245, 1530 and 1695 cm^−1^ are characteristic of polyurethanes and are due to valence vibrations within the urethane group, e.g., at 1695 cm^−1^, the vibration of the CO double bond [36]. The peak expressions in the formulas with salt and solvent are smaller the higher the additive amount is, which can be explained by the lower weight fraction of the polyurethane matrix. The small belly at about 2400 cm^−1^ might be CO_2_. This is formed especially when isocyanate reacts with water, which may have been additionally brought into the mixture by salt and solvent (both hygroscopic) [38]. Nevertheless, the slightly higher water content in the formulas with additives compared with the matrix is not reflected in the curves. Another possibility is that the atmospheric correction of the spectrometer did not have the same effect on both samples.

### 3.7. Demonstration in ECD

**Figure 16:**
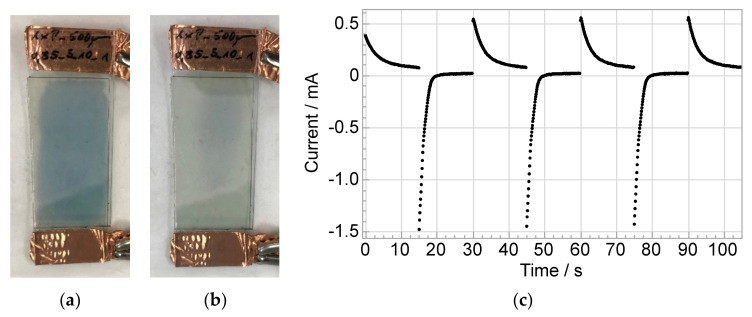
Electrolytes’ (formula 0.85_5_10) function demonstrated in an ECD (2 × 4 cm^2^ active area) with (a) the bleached state at +2.5 V and τ_v, bleached_ = 74.3% and (b) the colored state at −1.5 V and τ_v, colored_ = 64.0% and (c) the corresponding chronoamperogram of five cycles after conditioning; OH/NCO ratio/wt% salt/wt% solvent.

To demonstrate the function of the researched electrolyte, an exemplary ECD was manufactured and conducted with the electrolytes’ formula 0.85_5_10. To achieve a proper coloring effect a conditioning was necessary. Figure 16 shows the resulting switching states and the corresponding chronoamperogram of five cycles with −1.5/+2.5 V and 30 s cycle time. The visible light transmittance was τ_v, bleached_ = 74.3% in the bleached and τ_v, colored_ = 64.0% in the colored state (measured in cycle 5). The visible light transmittance change was accordingly Δτ_v_ = 10.3% and the corresponding contrast ratio (CR = τ_v, bleached_/τ_v, colored_) was CR = 1.16. At the ECDs edges, areas without electrolyte are visible that did not color. Further edge effects, in particular due to cracks in the metal oxide layers caused by the cutting of the films, could contribute to this. In the bleached state at +2.5 V the ECD locally still shows a slight coloring, which indicates that more time for complete bleaching is necessary. Electrolyte thickness was around 50 µm.

## 4. Conclusions

The additives amount and the matrix stoichiometric ratio affected the curing behavior and curability. So, the possible variation of additives amount and matrix stoichiometric ratio is limited. The salt content had almost no influence on the measured properties in the chosen experimental space (5 wt%/10 wt%). To further decrease the amount of salt for economic reason and possibly find an optimum regarding the ion conductivity seems reasonable. Solvent content had a great influence on ion conductivity and mechanical properties. An understoichiometric ratio of the polyurethane matrix (0.85) increased not only the ion conductivity and the mechanical flexibility, but also the optical properties, in a negative manner. Because the final thickness of the electrolyte’s layer in the ECD is below 100 µm, the observed slight yellowing and transmittance reduction might be acceptable. The best specific ion conductivity with 10^−5^ S/cm was reached with an understoichiometric ratio of 0.85, 5 wt% salt and a high solvent content of 30 wt%, even if the application in ECDs needs to be verified, because the high temperatures necessary for curing could be problematic regarding the brittle metal oxide layers that possibly cannot resist the plastics thermal extension. However, even the formula with 0.85 matrix ratio and low salt (5 wt%) and solvent (10 wt%) content that is curable at 40 °C had a sufficient ion conductivity of 3.3 × 10^−7^. Further investigations in order to optimize the conductivity by further varying the stoichiometric ratio and solvent content (considering the curability limits) and reducing salt content are promising.

Concluding, due to its high flexibility and transmittance, color neutrality and sufficient ion conductivity, the application of the researched electrolyte in ECDs might be suitable.

## Figures and Tables

**Table 1:** Electrolyte formulations used and their curability at 40 °C and 100 °C.

	Formula Abreviation	Stoichiometric Ratio of Polyurethane Matrix (OH/NCO)	Parts by Weight of Salt (LiTFSi)/wt%	Parts by Weight of Solvent (Propylene Carbonate)/wt%	Curable at 100 °C	Curable at 40 °C	
a	1_0_0	1	0	0	yes	yes	Screening Experiment
b	1_5_10	1	5	10	yes	yes	
c	1_10_10	1	10	10	yes	yes	
d	1_5_20	1	5	20	yes	yes	
e	1_10_20	1	10	20	yes	yes	
f	0.85_5_10	0.85	5	10	yes	yes	
g	1.15_5_10	1.15	5	10	yes	(yes)	
h	0.85_10_20	0.85	10	20	yes	no	
i	0.85_5_30	0.85	5	30	yes	no	
j	1_15_30	1	15	30	yes	no	
k	1_1.5_4	1	1.5	4	yes	yes	

**Table 2:** No significant influence of the manufacture and cure atmosphere on the electrolytes’ matrix material polyurethane.

	Mixture	Atmosphere		Tensile Strength/Pa		Elongation at Break/%		Water Content/%	
		Manufacturing	Curing	Mean	Standard Deviation	Mean	Standard Deviation	Mean	Standard Deviation
a	1_0_0 *	air	air	3.09	0.45	108.72	19.01	0.094	0.021
		N2	N2	3.19	0.40	107.73	16.31	0.049	0.026
		N2	50 mbar	-	-	-	-	0.047	0.007

* OH/NCO ratio/wt% salt/wt% solvent.

## Data Availability

Not applicable.
